# Skeletal Muscle an Active Compartment in the Sequestering and Metabolism of Doxorubicin Chemotherapy

**DOI:** 10.1371/journal.pone.0139070

**Published:** 2015-09-24

**Authors:** Sergio Fabris, David A. MacLean

**Affiliations:** 1 Biomolecular Sciences, Laurentian University, Ontario, Canada; 2 Divison of Medical Sciences, Northern Ontario School of Medicine, Sudbury & Thunder Bay, Ontario, Canada; University of Louisville School of Medicine, UNITED STATES

## Abstract

Doxorubicin remains one of the most widely used chemotherapeutic agents however its effect on healthy tissue, such as skeletal muscle, remains poorly understood. The purpose of the current study was to examine the accumulation of doxorubicin (DOX) and its metabolite doxorubicinol (DOXol) in skeletal muscle of the rat up to 8 days after the administration of a 1.5 or 4.5 mg kg-1 i.p. dose. Subsequent to either dose, DOX and DOXol were observed in skeletal muscle throughout the length of the experiment. Interestingly an efflux of DOX was examined after 96 hours, followed by an apparent re-uptake of the drug which coincided with a spike and rapid decrease of plasma DOX concentrations. The interstitial space within the muscle did not appear to play a significant rate limiting compartment for the uptake or release of DOX or DOXol from the tissue to the circulation. Furthermore, there was no evidence that DOX preferentially accumulated in a specific muscle group with either dose. It appears that the sequestering of drug in skeletal muscle plays an acute and important role in the systemic availability and metabolism of DOX which may have a greater impact on the clinical outcome than previously considered.

## Introduction

Doxorubicin (DOX) is one of the most widely used chemotherapeutic agents for the treatment of solid tumors and hematological malignancies. DOX induced cytotoxicity has been attributed to the inhibition of DNA replication and RNA transcription via DNA intercalation, inhibition of topoisomerase II (TOP2A) resulting in TOP2-mediated DNA damage, the formation of reactive oxygen species and hydrogen peroxide [1–3]. Although effective, DOX is strongly limited by a dose-dependent and cumulative cardiotoxic side effect which has been primarily attributed to its metabolite Doxorubicinol (DOXol) [4, 5]. DOXol is ten times more cytotoxic than DOX and highly cardiotoxic as a result of its potent inhibition of the sarcoplasmic reticulum calcium pump, the sarcolemmal Na^+^/K^+^ pump and the mitochondrial F_0_F_1_ proton pump [4, 6]

To date, much of the research remains focused on reducing the cardiotoxic effect related to the use of DOX. However, recent studies have reported that the exposure of skeletal muscle to DOX causes significant physiological effects including a progressive decline in muscular function [7], a decline in maximal twitch force and rate of force generation [8] as well as increased muscular fatigue [9]. Cellular dysfunction has been shown to be related to a significant increase in mitochondrial reactive oxygen species [10]. In addition, upregulated autophagy and apoptotic pathways are believed to be primarily responsible for the DOX-induced decrease in muscle mass [8, 11, 12]. Despite the growing evidence suggesting a DOX related myotoxicity, the effect of DOX on skeletal muscle metabolism and function has received little attention.

In skeletal muscle, the interstitial space represents an important compartment located between the vasculature and the tissue. It has been shown to play a functional role in the regulation and integration of various metabolic substances [13–15]. Microdialysis is used to directly measure these substances in the interstitial space *in vivo*. The microdialysis technique, first described by Delgado *et al*. [16], is based on the principle of simple diffusion through a semipermeable membrane which allows for the direct measurement of compounds, including DOX and DOXol, located within the interstitial space directly at the tissue level. To date, not only has DOX and DOXol not been thoroughly examined in the vascular, interstitial and muscular compartments, but the relationship between these compartments has never been investigated. Therefore, the purpose of the current study was to investigate the accumulation of DOX and DOXol in skeletal muscle and examine the relationship between the muscular, interstitial and vascular compartments following administration of DOX in the rat. Overall, our results clearly indicate that DOX and DOXol is sequestered in the skeletal muscle and that there were no differences in the sequestering of the drug or its metabolite in muscle groups containing predominantly fast or slow muscle fibers. Therefore, it appears that skeletal muscle plays an acute and important role in the systemic availability and metabolism of DOX.

## Results

### Plasma DOX

**Fig 1:**
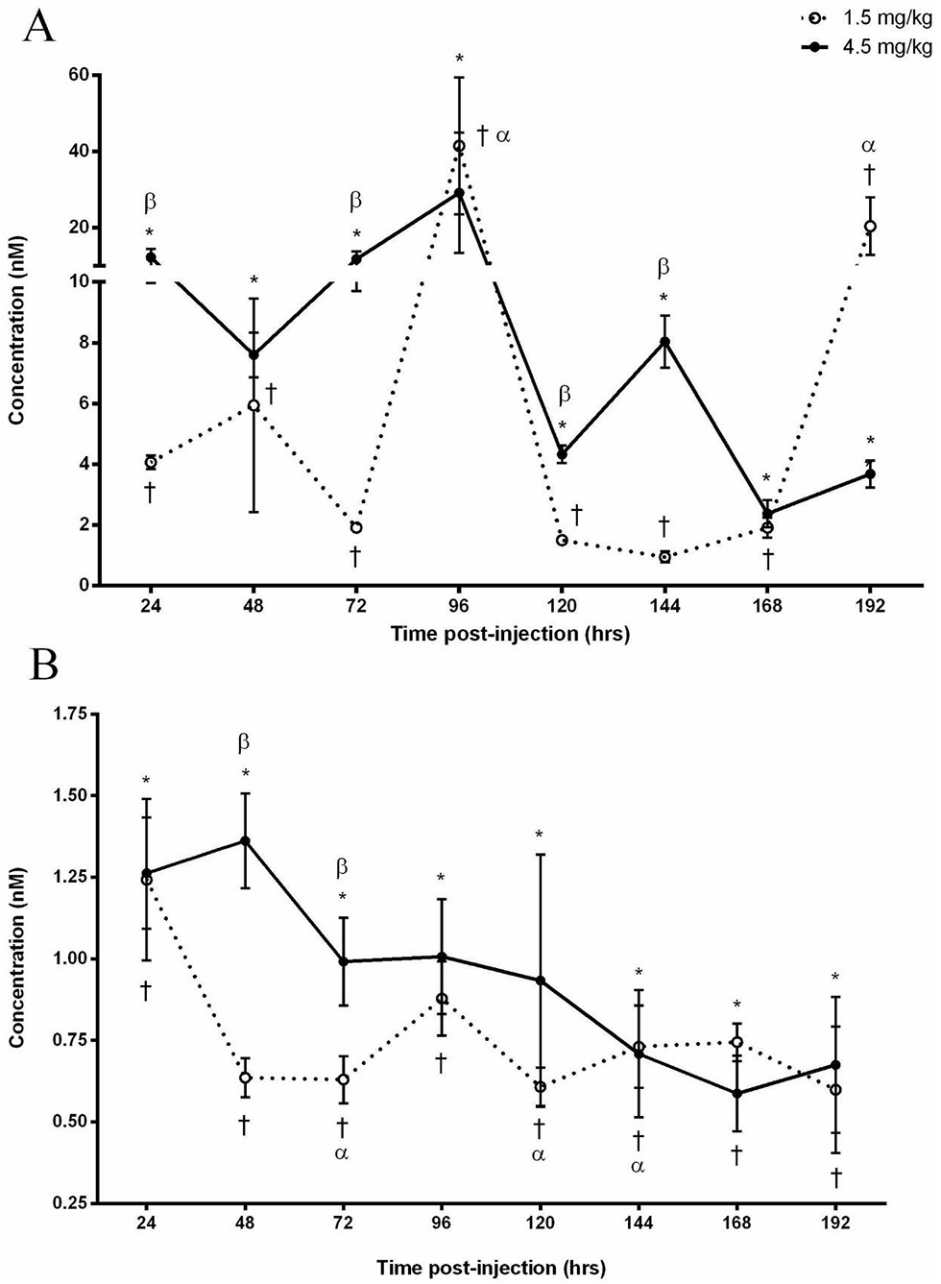
Doxorubicin (A) and Doxorubicinol (B) concentrations in the arterial plasma following the IP administration of 1.5 or 4.5 mg kg-1 Doxorubicin. (A) Significance compared to baseline for the 1.5 mg kg-1 dose is denoted by ✝ and 4.5 mg kg-1 by *. α Denotes an increase (P<0.05) compared to 24, 48, 72, 120, 144 and 168 hours as a result of the 1.5 mg kg-1 dose. β Denotes difference (P<0.05) between administered doses. (B) Significance compared to baseline for the 1.5 mg kg-1 dose is denoted by ✝ and 4.5 mg kg-1 by *. α Denotes a decrease (P<0.05) compared to 24 hours. β Denotes difference (P<0.05) between administered doses.

The administration of 1.5 mg kg-1 DOX elevated (P<0.05) arterial concentrations of DOX throughout the experiment, with the highest concentration observed after 96 (41.4±17.9 nM) and 192 hours (20.3±7.5 nM) post injection as compared to baseline. Similarly, circulating concentrations were elevated (P<0.05) throughout the experiment following the administration of 4.5 mg kg-1 DOX. When compared to the 1.5 mg kg-1 dose, the concentration of DOX was greater (P<0.05) following the 4.5 mg kg-1 dose after 24 (300±77%), 72 (609±119%), 120 (288±0%) and 144 (845±147%) hours (Fig 1A).

### Plasma DOXol

Following the administration of 1.5 mg/kg, DOXol was measurable (P<0.05) after 24 hours (1.24±0.25 nM) and remained measurable throughout the experiment. In comparison to the initial concentration observed after 24 hours, the levels were decreased (P<0.05) by 51±10%, 49±11% and 48±12% after 72, 120 and 144 hours respectively. The 4.5 mg kg-1 dose resulted in an initial DOXol increase (P<0.05) after 24 hours (1.26±0.17 nM) which remained stable for the remainder of the experiment. Despite the fluctuation difference of DOXol in both doses, the 4.5 mg kg-1 dose was observed to be elevated (P<0.05) after 48 (213±7%) and 72 (157±6%) hours as compared to the 1.5 mg kg-1 dose (Fig 1B).

### Skeletal Muscle DOX

**Fig 2:**
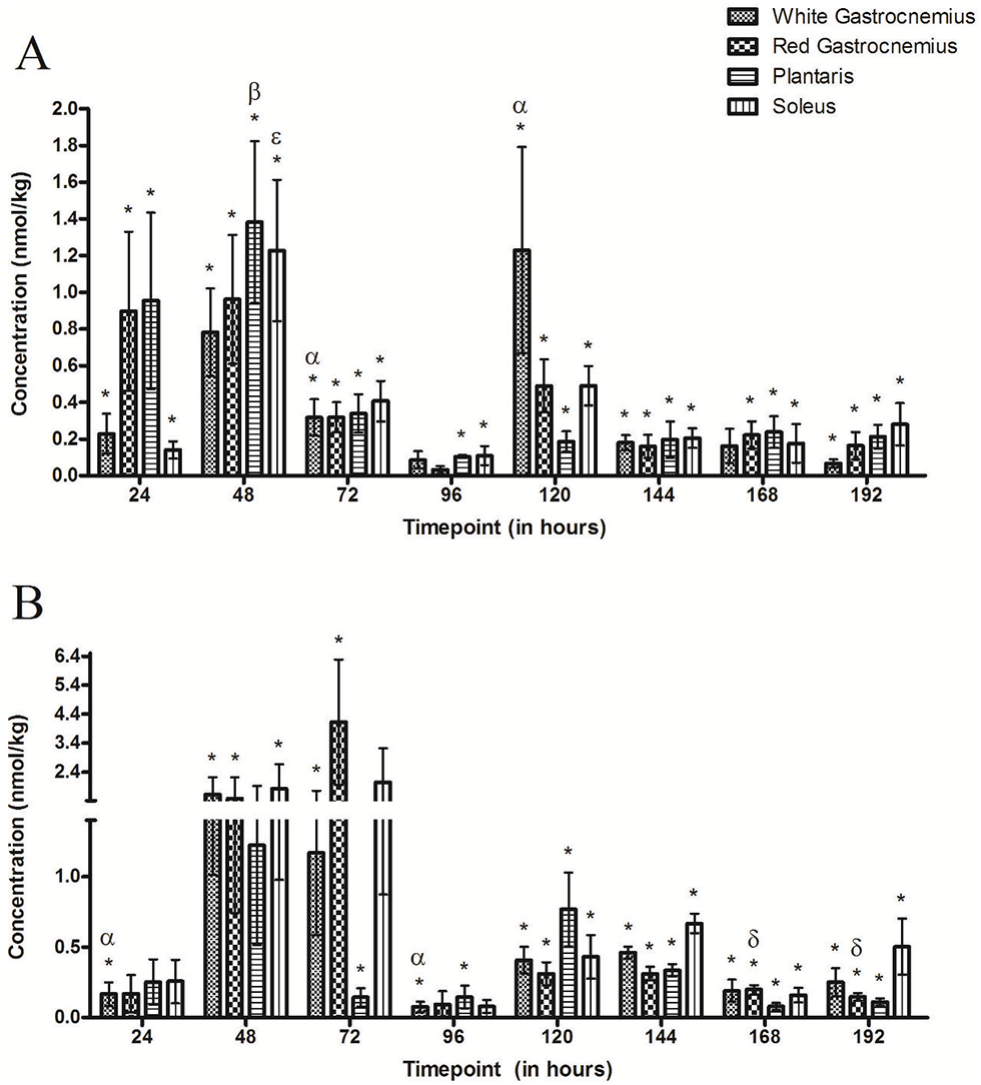
Doxorubicin concentrations in skeletal muscle groups following the IP administration of (A) 1.5 mg kg-1 or (B) 4.5 mg kg-1 of Doxorubicin. (A) * Denotes significance when compared to baseline for all muscle groups. α Indicates the highest points of accumulation of DOX (P<0.05) in the white gastrocnemius, β Indicates the highest point of accumulation (P<0.05) in the plantaris and ε Indicates the point of greatest accumulation of DOX (P<0.05) in the soleus. (B) * Denotes significance when compared to baseline for all muscle groups. α Denotes a decrease (P<0.05) compared to 48 hours in the white gastrocnemius and δ denotes a significant decrease compared to 72 hours in the red gastrocnemius. There are no significant differences between muscle types at any time point in either administered doses.

Following the 1.5 mg kg-1 dose, DOX was detectable in the WG at each time point (P<0.05) with the exception of 96 and 168 hours. Additionally, these concentrations were elevated (P<0.05) after 48 (0.78±0.24 μmol/kg), 72 (0.32±0.1 μmol/kg) and 120 (1.23±0.56 μmol/kg) hours post-injection (Fig 2A). The administration of 4.5 mg kg-1 dose resulted in measurable (P<0.05) DOX concentrations at each time point. When compared to 48 hours (1.62±0.61 μmol/kg), 10 and 20-fold decreases (P<0.05) were observed after 24 and 96 hours, respectively (Fig 2B).

With the exception of the 96 hour time point, the concentration of DOX was measurable (P<0.05) RG at each time point of the experiment following the administration of 1.5 mg kg-1 dose with an initial tissue concentration of 0.90±0.43 μmol/kg after 24 hours (Fig 2A). DOX was measurable (P<0.05) at the 48, 72, 120, 144, 168 and 192 hour time points following the administration of the 4.5 mg kg-1 dose. The concentration of DOX significantly decreased 200-fold after 168 hours and 26-fold after 192 hours when compared to the 72 hour (4.13±2.16 μmol/kg) time point (Fig 2B).

Subsequent to the 1.5 mg kg-1 dose, DOX concentrations in the PL were measurable at each time point and were elevated (P<0.05) to 1.38±0.44 μmol/kg after 48 hours. Concentrations subsequently decreased (P<0.05) after 120 (726±0%), 144 (690±6%), 168 (575±0.2%) and 192 hour (675±1%) in comparison to the 48 hour time-point (Fig 2A). The administration of 4.5 mg kg-1 resulted in measurable concentrations of DOX after 72 hours (0.14±0.06 μmol/kg, P<0.05) which remained consistent for the remainder of the experiment (Fig 2B).

The administration of 1.5 mg kg-1 DOX resulted in measurable concentrations of DOX within the SOL at each time point with the highest (P<0.05) concentration (1.23±0.39 μmol/kg) occurring after 48 hours (Fig 2A). DOX was measurable (P<0.05) in the SOL at each time point with the exception of the 24, 72 and 96 hour time points following the 4.5 mg kg-1 dose (Fig 2B).

Interestingly, no consistent differences between intracellular DOX concentrations were observed throughout the course of the experiment when comparing muscle groups at either the administration of 1.5 or 4.5 mg kg-1 dose. Furthermore, there were no significant dose-dependent differences in DOX concentrations at any time point when comparing administered doses.

### Skeletal Muscle DOXol

**Fig 3:**
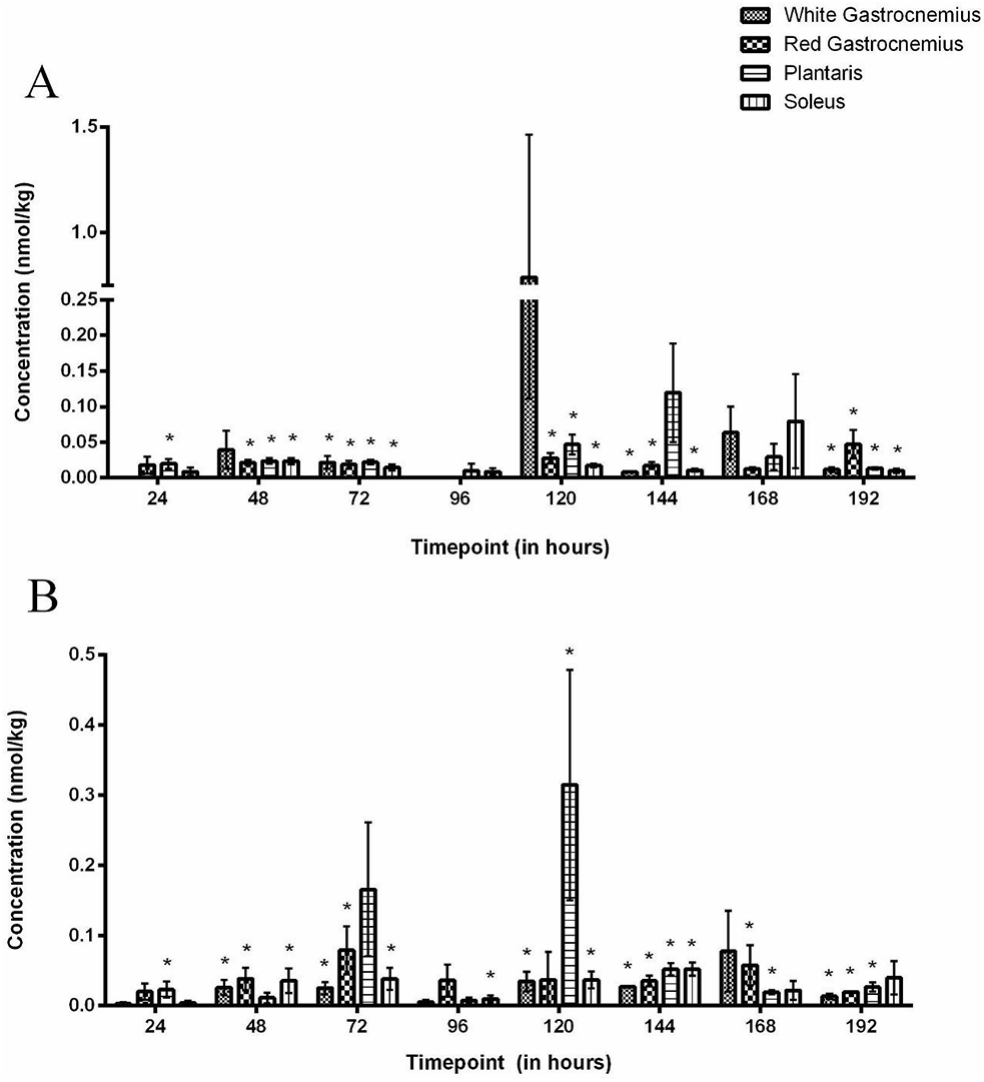
Doxorubicinol concentrations in skeletal muscle groups following the IP administration of (A) 1.5 mg kg-1 or (B) 4.5 mg kg-1 of Doxorubicin. * Denotes significance when compared to baseline for all muscle groups. There are no significant differences between muscle types at any time point in either administered doses.

DOXol was measurable (P<0.05) in the WG after 72, 144 and 192 hours as a result of the administered 1.5 mg kg-1 dose (Fig 3A). The administration of 4.5 mg kg-1 dose resulted in measureable (P<0.05) DOXol concentrations after 48, 72, 120, 144 and 192 hours (Fig 3B).

The administration of 1.5 mg kg-1 dose resulted in measurable DOXol concentrations (P<0.05) in the RG after 48, 72, 120, 144 and 192 hours (Fig 3A). The 4.5 mg kg-1 dose resulted in measurable (P<0.05) tissue concentrations of DOXol at 48, 72, 144, 168 and 192 hours (Fig 3B).

The 1.5 mg kg-1 dose resulted in measurable (P<0.05) concentrations of DOXol in the PL after 24, 48, 72, 120 and 192 hours (Fig 3A), whereas the 4.5 mg kg-1 dose resulted in measurable (P<0.05) levels of DOXol after 24, 120, 144, 168 and 192 hour (Fig 3B).

DOXol was measurable (P<0.05) in the SOL after 48, 72, 120, 144 and 192 hours following the 1.5 mg kg-1 dose (Fig 3A). Similarly, DOXol was measurable (P<0.05) after 48, 72, 96, 120 and 144 hours post-injection of the 4.5 mg kg-1 dose (Fig 3B).

Interestingly, there were no consistent differences between DOXol concentrations when comparing muscle groups following the 1.5 mg kg-1 or 4.5 mg kg-1 dose. Additionally, there were no significant dose-dependent differences in DOXol concentrations at any time point when comparing administered doses.

### Interstitial DOX

**Fig 4:**
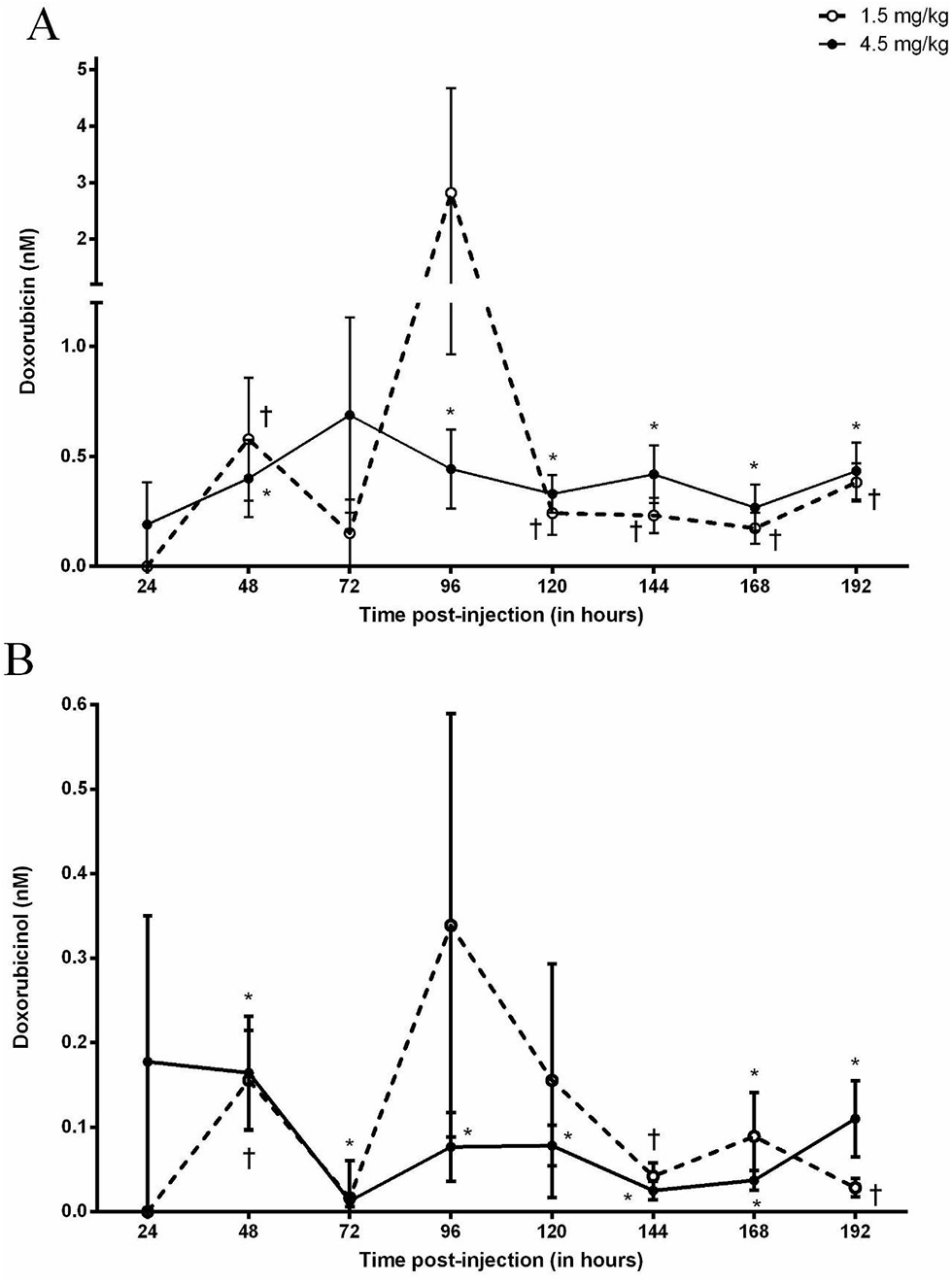
Doxorubicin (A) and Doxorubicinol (B) concentrations in the interstitial space of skeletal muscle following the IP administration of 1.5 or 4.5 mg kg-1 Doxorubicin. ✝ Designates significance compared to baseline for the 1.5 mg kg-1 dose. * Designates significance compared to baseline for the 4.5 mg kg-1 dose.

Following the 1.5 mg kg-1 dose, interstitial DOX concentrations were measurable (P<0.05) after 48 hours (0.58±0.28 nM) then decreased until 120 hours (0.24±0.1 nM; P<0.05) where levels remained stable for the remainder of the experiment. Similarly, as a result of the 4.5 mg/kg, DOX was measurable (P<0.05) after 48 hours (0.4±0.18 nM) and decreased until 96 hours (0.44±0.18 nM; P<0.05) where concentrations remained stable for the remainder of the experiment. There were no significant differences in interstitial DOX concentrations at any time point when comparing between administered doses (Fig 4A).

### Interstitial DOXol

Interstitial DOXol concentrations were measurable (P<0.05) after 48, 144 and 192 hours post-administration of the 1.5mg kg-1 dose. Subsequent to the 4.5 mg kg-1 dose, DOXol was measurable after 48 hours (0.16±0.07 nM) and remained stable for the remainder of the experiment. There were no significant differences between interstitial DOXol concentrations between doses (Fig 4B).

## Discussion

The focus of the current study was to investigate the accumulation of DOX and DOXol in skeletal muscle and examine the relationship between the muscular, interstitial and vascular compartments following the administration of DOX in the rat. A major finding in this study was the apparent role that skeletal muscle plays in the sequestering of DOX following its administration. This was made evident as plasma DOX levels were inversely proportional to skeletal muscle concentrations (data presented Figs 1 and 2). A static pool of DOX was established in the interstitial space which maintained in dynamic equilibrium between the vasculature and skeletal muscle which did not change over time or with an increase in dose. As such, the interstitial space does not appear to represent a rate limiting factor in the diffusion kinetics of the drug. Furthermore, the relationship between the three compartments was impartial to muscle groups predominantly composed of different fiber types. Hence, it appears that skeletal muscle plays an acute and important role in the systemic distribution of DOX and may have a greater impact on drug availability than previously considered.

DOX was administered IP in order to simulate previous studies that have examined the cardiotoxic effects of DOX in the rat [17–20]. A single low dose of DOX was used in order to minimize the drug-induced risk of cardiotoxicity. For example, previous studies have utilized a variety of concentrations and treatment regimens documenting significant cardiomyocyte damage. Iqbal et al. [21] administered a single 20 mg kg-1 dose IP and observed the induction of acute cardiotoxicity after 48 hours and up to 7 days post administration. A study by Nagi et al. [22] reported that a single dose of 15 mg kg-1 IP induced cardiotoxicity after 24 and 48 hours and Jensen et al. [18] noted that a 10 mg kg-1 or less total dosage did not cause cardiac failure. Although Arola et al. [23] reported minor cardiomyocyte apoptosis after 3 days following the injection of 1.5 mg kg-1 and 5 mg/kg, it remains unclear whether this significantly altered cardiac performance. Cumulative doses have been studied where 1 mg kg-1 (IP) was administered 15 times over 3 weeks resulted in cardiac dysfunction [17] and 1.5 mg kg-1 weekly for 9 weeks resulted in severe cardiomyopathy [20]. What these studies have in common is the focus on the long term effects of DOX where, to our knowledge, little or no attention has been given to the early stages of DOX accumulation in skeletal muscle. As such the current study investigated DOX concentrations 24 hours sequentially up to 192 hours. With the aforementioned factors taken into consideration, the concentrations used in this study provided a dose-related experimental contrast while minimizing the drug-induced risk of cardiac dysfunction associated with the higher doses. Additionally, the time frame allows for the examination of acute DOX accumulation in skeletal muscle as well as the dynamic between the circulation and the interstitial space within the skeletal muscle.

This study represents the first time that sequential measurements of plasma DOX concentrations have been made for 8 days (192 hours) after IP administration. Despite the threefold increase in administered dose from 1.5 mg kg-1 to 4.5 mg/kg, a stable circulating concentration of DOX was maintained throughout the 8 days with the exception of a spike in concentration after 4 days (96 hours). This stable concentration may be attributed to the enzymatic breakdown of DOX once in the circulation. It is well known that DOX is enzymatically reduced to a number of metabolites [24]. The reduction of DOX to its major metabolite, DOXol [25] by way of carbonyl reductase-1 [26], carbonyl reductase-3 [27] or aldo-keto reductases [28] is of particular clinical importance as it is 10 times more cytotoxic than DOX [29] and has been identified to be responsible for the cardiotoxic effect related to DOX therapy [4]. DOXol concentrations in the plasma did not have the same degree of variability within the dose groups or degree of dose response as that observed in the DOX concentrations. What is interesting, however, is that despite the increase in DOXol concentrations between doses after 48 and 72 hours, concentrations remained constant for the remainder of the experiment indicating that the breakdown of DOX to DOXol does not complement the increase in administered dose. This would indicate that a threshold in the enzymatic breakdown of DOX to DOXol may exist and a maximal rate was achieved 24 hours after drug administration which resulted in a constant circulating concentration of the cytotoxic metabolite. The persistent circulation of DOX represents a significant clinical importance as the repeated administration of DOX may further increase circulating concentrations of the drug and its toxic metabolite above that established by the initial dose. While this may be beneficial in assuring the delivery to the tumor, increasing the drug and, as a result, its toxic metabolite may have a greater impact on healthy tissue.

The current study proposes that the persistent circulating concentration of DOX is attributed to the acute sequestering of the drug in the skeletal muscle which is primarily responsible for the timely release of the drug back into the circulation. Previous studies have focused on the accumulation of DOX in the heart, liver, spleen, lung, brain and kidney [30–34]. However very few have investigated the accumulation in skeletal muscle [7, 35, 36]. The accumulation of DOX was observed in the muscle at each time point regardless of the administered dose. What was most surprising was the consistent efflux of DOX at 96 hours which was quickly followed by a re-accumulation after 120 hours with both doses. The rapid elimination of DOX from the skeletal muscle coincides with the notable increases seen in plasma as previously discussed. To our knowledge no mechanisms to date have been identified that may explain these results, although these data may indicate an active survival mechanism by the skeletal muscle in order to export DOX from the cytosol once a toxic concentration has been attained. As previously mentioned, a constant rate of DOX breakdown from the circulation may have been established after 24 hours of drug administration. As such, the sequestering of DOX in skeletal muscle may occur to assist in minimizing the toxic circulating concentrations while being subjected to the rate limiting enzymatic breakdown by the liver. It is also possible that after approximately 72 hours the sequestration of DOX into the skeletal muscle by way of passive diffusion [37], a threshold limit may have been achieved. The subsequent transcription of transport proteins such as RALBP1 [38] and/or P-glycoprotein [39] required for the active export of DOX may be initiated resulting in the efflux of DOX back into the circulation. The opportunistic release of drug back into the circulation and subsequent breakdown reduces toxicity both at tissue and systemic level. The apparent re-uptake of DOX into the skeletal muscle tissue may be a function of the rapid reintroduction of DOX into the circulation and resulting diffusion back into the tissue. DOXol was measurable in skeletal muscle up to 8 days, regardless of the administered dose. Although quantifiable, the accumulation of DOXol was minimal up to 96 hours which corresponds to the aforementioned metabolic breakdown of DOX. To our knowledge, data regarding the accumulation of DOXol in skeletal muscle remains limited [40–42] and the mechanisms involved in the apparent accumulation of DOXol in the skeletal muscle remains poorly understood. It may be possible that DOXol enters the cell by passive diffusion, in a similar process as that of DOX. In support of this notion, the constant circulating concentration of DOXol that was established after 96 hours would explain the increased accumulation in skeletal muscle. It is also possible that the accumulated concentration of DOX in skeletal muscle is broken down to DOXol at a steady rate by the presence of carbonyl reductase-1 resulting in a constant rate within the tissue [26, 43]. Given the well-established cytotoxic nature of DOXol, more research is necessary to understand the role that skeletal muscle plays in the accumulation and metabolism of this metabolite. It should be noted that the route of administration as well as the inherent individual differences of drug uptake from the gut may have contributed to these results. Alternative routes such as IV may yield a distinct contrast in concentrations between doses. Furthermore, as a limitation of the experimental procedures, these data are not paired as the same measurements were not made from the same rat over the course of the experiment (192 hours). Skeletal muscle represents approximately 40% of the body mass and constitutes an important compartment which appears to play a major role in the sequestering of DOX. The resulting accumulation of the drug in the muscle may have a more immediate impact on the tissue than previously believed. More concerningly, the apparent sequestering may significantly limit the amount of drug intended for the target tissue (i.e. tumor) upon repeated administration while inadvertently increasing concentrations while unnecessarily elevating DOXol in the healthy tissue.The interstitial space within the skeletal muscle did not appear to represent a significant rate limiting compartment for the uptake or release of DOX or DOXol from the tissue to the circulation irrespective of the dose. Interstitial DOX and DOXol concentrations were stable throughout the experiment as well as comparable between the two doses. This suggests that the rate of appearance of DOX and DOXol in the interstitial space from the vasculature appears to be matched by the rate of disappearance of the drug and its metabolite from the interstitial space into the muscle. A dynamic equilibrium appears to form resulting in no net accumulation in the interstitial space. This is further supported by the finding that the reciprocal efflux of DOX and DOXol from the muscle back into the plasma at 96 hours did not result in any significant accumulation of these compounds in the interstitial space, suggesting that the dynamic equilibrium is sustained in both directions. Additionally, these data demonstrate that the microdialysis technique is an effective tool for the *in vivo* analysis of drug concentrations within the extracellular fluid of skeletal muscle, and as such, may be an important tool in the assessment of drug delivery kinetics.

Muscle groups which differ in fiber type distribution were examined to further investigate the factors involved in the accumulation of DOX and DOXol within skeletal muscle. The SOL predominantly consists of ~97% of Type I fibers whereas the WG is ~88% Type IIB fibers [44]. The RG (~39% Type I, ~30% Type IIA) and PL (~45% Type IIX, ~21% Type IIA) are considered as a mixed based on the distribution of fiber types [44]. Interestingly, there was no evidence that DOX preferentially accumulated in a specific muscle group at either the 1.5 mg kg-1 or 4.5 mg kg-1 dose. Additionally, no differences in DOX concentrations were observed when comparing levels between the two doses. It has previously been shown that once in the cytosol DOX has a high affinity for cardiolipin [45], an important phospholipid expressed in the mitochondrial membrane. It would be expected that a preferential accumulation of DOX would occur in the oxidative Type I muscle as it relies heavily on mitochondrial activity as compared to the Type II glycolytic muscle. Though, the current data supports the contention proposed by Anderson *et al*. [46] who has reported a high degree of variability of the accumulation of DOX in the mitochondria. It is well known that the capillary network within the skeletal muscle differs greatly between fiber types where the capillary to fiber ratio is significantly higher in Type I muscle compared to that of Type II [47, 48]. In the current study, it does not appear that the difference in perfusion of the tissue plays a role in the accumulation of DOX in the skeletal muscle. The data in the current study represent basal muscle conditions as there was minimal muscle exertion over the course of the experiment. Muscle stimulation or exercise may expose fiber type differences in the accumulation of DOX and, as such, further study is required to better explore this possibility. There was some individual variation in DOXol accumulation amongst the muscle groups but overall there was no difference between muscle groups regardless of the administered dose. As previously noted, the mechanisms responsible for the influx and efflux of DOXol from skeletal muscle has not yet been elucidated and even less is known about the commonalities shared between fiber types leading to the results seen in this study. Considering the resemblance of data between DOXol and DOX, it is within reason to believe that muscle fiber types do not affect DOXol accumulation in basal conditions. Further study is required to explore the possible role that increased muscle use may have on accumulation.

Overall, this study clearly shows that skeletal muscle plays an important role in the metabolism of DOX and may significantly alter its therapeutic impact. The rapid and sustained accumulation of the drug in the muscle as a result of a single injection may initiate factors leading to the characteristic dysfunction previously described. Furthermore, the apparent sequestering of the drug in the muscle effectively reduces systemic concentrations and favors metabolic breakdown. In turn, this decreases the drug intended for the tumor severely reducing the therapeutic impact prompting repeated doses typical of chemotherapy regimens. This represents a therapeutic and clinical paradox: The repeated administration of the drug increases the exposure of the tumor to the chemotherapy, while the inherent accumulation of the drug in skeletal muscle exacerbates the initial detrimental effects, ultimately reducing the clinical outcome. The possible manifestation of skeletal muscle toxicity before that of a cumulative and dose-dependent cardiotoxicity may have serious implications in the clinical assessment and modulation of Doxorubicin chemotherapy.

## Materials and Methods

### Animals

All of the experimental procedures in the study were approved by the Laurentian University Animal Care Committee. Male Sprague-Dawley rats (n = 102, 434±8 g) were obtained from Charles River Laboratories (Senneville, QC) and were housed according to Standard Operating Procedures and Policies for the Housing and Environmental Enrichment of Rodents at the Laurentian University Animal Care Facility. Experiments began after a one week acclimation period.

### Pre-experimental procedures

#### Experimental groups

**Fig 5:**
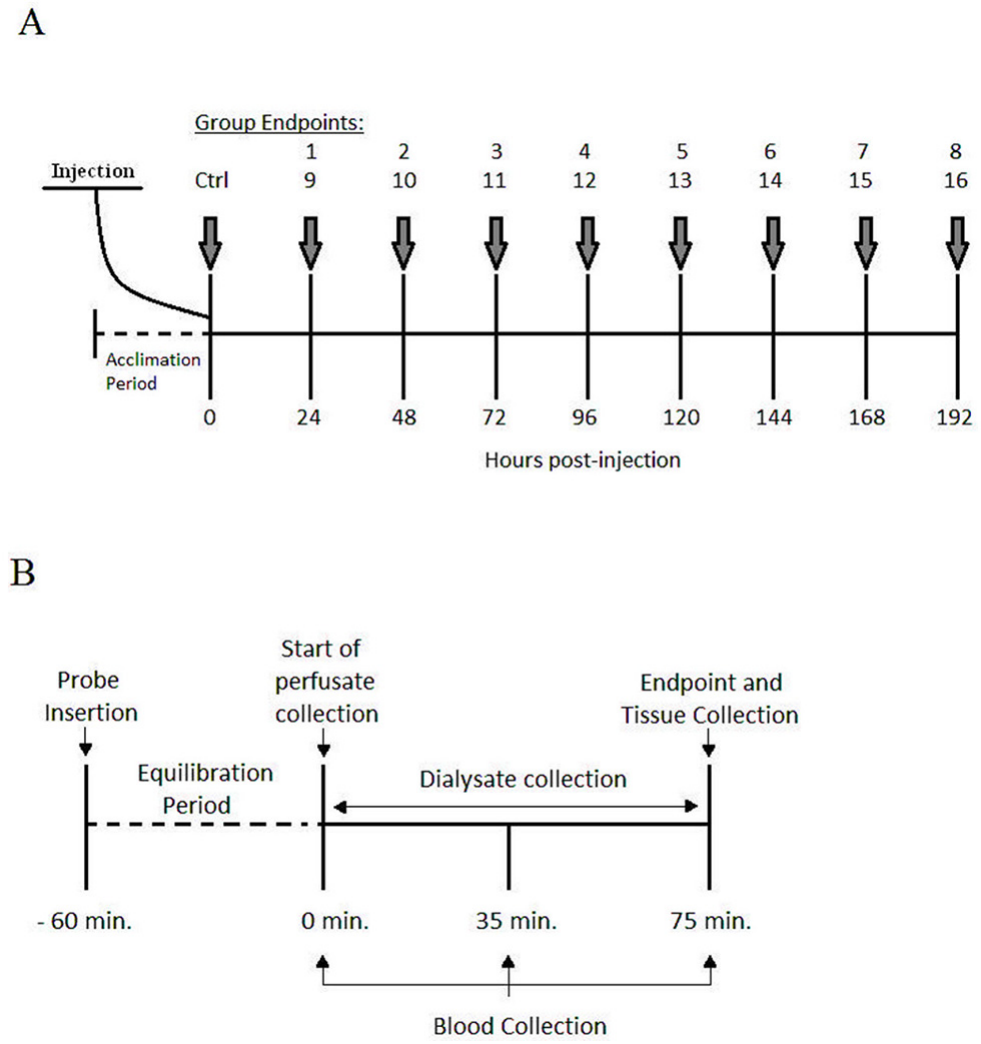
Organization of experimental groups and experimental procedures. (A) Schematic representation of the experimental groups receiving an injection of 1.5 mg kg-1 dose (groups 1–8) or 4.5 mg kg-1 (groups 9–16) of DOX IP. A sham injection is administered to the control group (Ctrl). (B) Representation of the experimental proceedings for dialysate, blood and tissue collection following microdialysis probe insertion and carotid artery cannulation.

Rats were injected with Doxorubicin (Doxorubicin Hydrochloride. Pfizer, Canada) at a dose of 1.5 mg kg-1 (Group 1 to 8) or 4.5 mg kg-1 (Group 9 to 16), administered intraperitoneally (IP). A sham injection of saline solution was administered to a control group (IP, n = 6). Once administered, rats were randomly grouped into experimental endpoints (n = 6) of 24, 48, 72, 96, 120, 144, 168 or 192 hours post-injection (Fig 5A). This dose was selected in order to examine the acute effects of the drug on skeletal muscle while minimizing the potential for cardiotoxicity.

#### Microdialysis probes

A complete description of the construction of the microdialysis probes has previously been outlined [49]. Briefly, microdialysis fibers were constructed using Spectra Microdialysis fibers (Spectrum, VWR International) with a molecular weight cut-off of 13 kDa and Polyimide-100II tubing (MicroLumen, Tampa FL) cut to 9.5 and 5 cm lengths. The ends of the fiber were inserted 1 cm into the hollow polyamide tube and glued. The exposed portion of the fiber (diffusible) between the polyamide tubes measured 1.0 cm in length.

### Experimental procedures

#### Animals

On the day of the experiment, the rats were placed into an induction chamber and anaesthetized using an EZ-150 vaporizer unit (EZ-Anesthesia, Euthanex Corporation, Palmer, PA), which blends oxygen (100% O_2_ Praxair, Sudbury, ON) and isoflurane (5%). Once anaesthetized, the rats were removed from the chamber and placed onto a heated pad while its nose was placed in to a nosepiece supplying a continuous delivery of oxygen and isoflurane (2–2.5%). The animals remained under anaesthesia throughout the experiment and the plane of anaesthesia was routinely checked by toe pinches. Heart rate and oxygen saturation were monitored throughout the experiment using a pulse oximeter (SurgiVet) attached to the base of the tail. Body temperature (between 36–37°C) was maintained using a heated surgical bed (EZ-Anaesthesia) and a heating lamp. Following the completion of the experiment the animals were euthanized by decapitation.

#### Probe insertion

**Fig 6:**
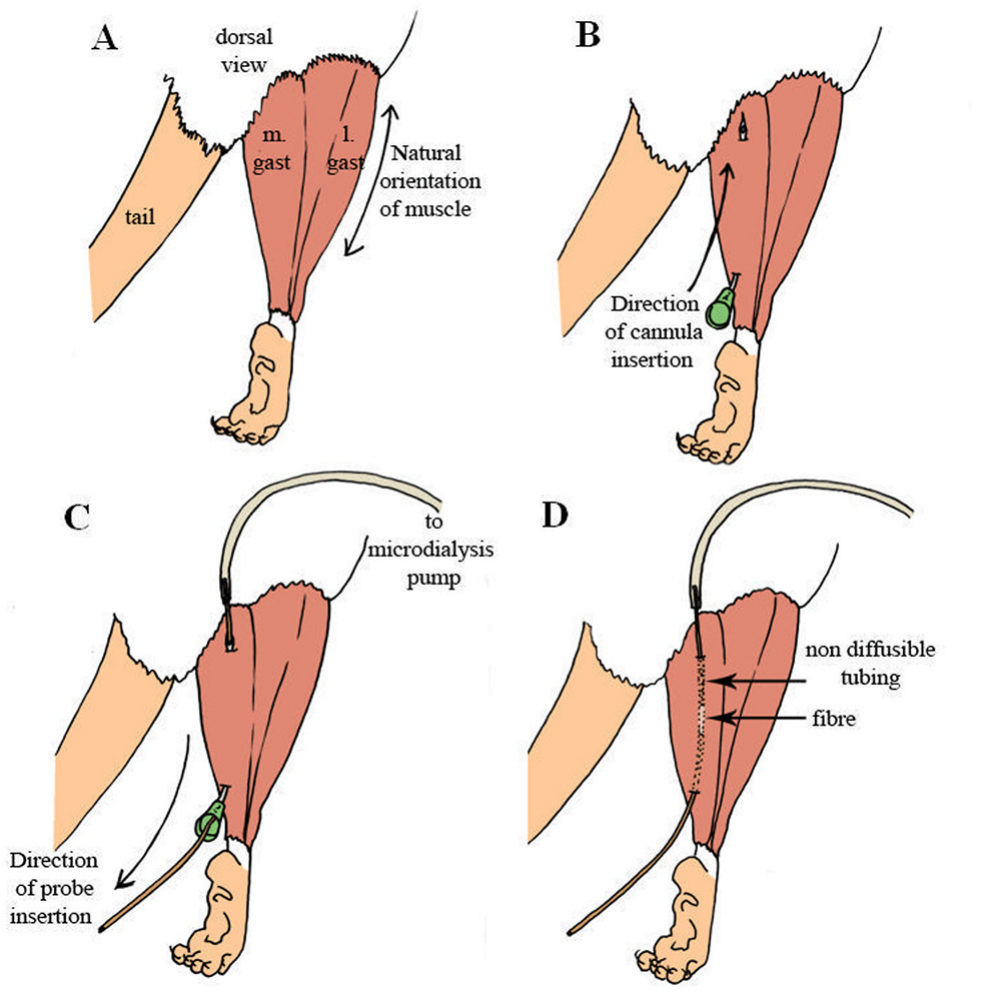
Experimental procedure for microdialysis probe insertion. (A) Exposing the medial and lateral gastrocnemius muscle. (B) Insertion of 21 gauge cannula. (C) Microdialysis probe insertion guided by the cannula. (D) Location of the diffusible membrane within the muscle.

Using curved dissecting scissors, incisions were made at the base of the leg and the skin was manually pulled back until the entire leg was exposed (Fig 6A) and then the excess skin was removed. A 21 gauge curved cannula was inserted anteriorly into the muscle along the muscle’s natural fibre orientation (Fig 6B) and acted as a guide for the microdialysis probe. Once the cannula was in place, the 9.5 cm portion of the probe is inserted caudally through the cannula and extended past the foot (Fig 6C). As soon as the diffusible portion of the probe was oriented into the muscle, then the 5 cm portion of the probe was held in place while the cannula was retracted out of the tissue leaving the probe in place (Fig 6D).

#### Experimental protocol

Two microdialysis probes were inserted into the hind limb, one in the lateral gastrocnemius and one in the medial gastrocnemius muscle of the same limb (n = 2 probes per animal) as the opposite limb was used for muscle extraction. Following probe insertion, the fibers were perfused (model 102, CMA) at a rate of 3 μL/min with Ringer’s solution. It is recognized that probe insertion results in some cellular disruption therefore traditionally a 60 min. equilibration period prior to the initiation of the experiment was used to ensure that the external environment surrounding the probes had stabilized and all cellular damage had dissipated [50]. Subsequently, the skin and tissue covering the left carotid artery was removed. A 20 gauge cannula was inserted into the exposed artery and sutured in place. Heparinized saline (100 μL) was injected into the artery to maintain patency in preparation for blood sampling.

Following the equilibration period the experimental protocol was initiated (Fig 5B) where dialysate was collected for a period of 75 minutes in order to obtain sufficient volume that was required for analysis. Dialysate samples were collected in microcentrifuge tubes and immediately sealed to prevent evaporation and stored at -80°C until analysis. Arterial blood samples were collected during the collection of dialysate at 0, 35 and 75 minutes. Samples were spun at 15,000 rpm for 25 seconds, plasma was separated and stored at -80°C until analysis. Upon completion of dialysate and blood collection the plantaris (PL), soleus (SOL), medial gastrocnemius (WG) and lateral gastrocnemius (RG) muscles of the opposite leg used for microdialysis were extracted and inserted into cryogenic vials then flash frozen in liquid nitrogen and stored at -80°C until analysis.

### Sample analysis

#### HPLC

Quantification of DOX and DOXol were determined by HPLC analysis (Waters e2695, 2475 Fluorescence Detector, Waters Corporation) with an ACE CN column (Advanced Chromatography Technologies, UK). The mobile phase was composed of a 10mM sodium phosphate monobasic (pH 4.0) solution as well as a 100% acetonitrile solution and a volume of 90 μL was used for analysis. Detection was made by fluorescence (2475 Fluorescence Detector, Waters Corporation) at 480 nm excitation and 560 nm emission.

#### Interstitial DOX, DOXol

Quantification of interstitial DOX and DOXol concentrations where determined by HPLC analysis. The collected dialysate samples represent an ultra-clean filtrate and did not require any pre-analysis preparation. Dialysate and perfusate blanks as well as dialysate samples from control animals were used to confirm the quality and purity of the samples analyzed. Compounds were quantified by comparison with a standard curve (Doxorubicin Hydrochloride. Pfizer, CA; Doxorubicinol Citrate. TRC, CA).

#### Skeletal Muscle DOX, DOXol

Muscle samples were homogenized using a 67 μM potassium phosphate solution containing a 0.625 μM Daunorubicin (Daunorubicin hydrochloride, Sigma) internal standard, to a concentration of 125 mg muscle/mL of solution. Subsequently, 200 μL of 50:50 (v/v) 40% ZnSO_4_ and 100% methanol was added to 150 μL homogenate and vortexed for 2 minutes. Samples were then placed in the centrifuge at 13,000 rpm for 10 minutes and supernatant was used for HPLC analysis. Compounds were quantified by comparison with a standard curve and corrected by internal standard.

#### Plasma DOX, DOXol

For this analysis, a 133 μL of 50:50 (v/v) 40% ZnSO_4_ and 100% methanol containing 0.0625 μM Daunorubicin internal standard was added to 100 μL of plasma and vortexed for 2 minutes. Samples were then placed in the centrifuge at 13,000 rpm for 10 minutes and supernatant was used for HPLC analysis. Compounds were quantified by comparison with a standard curve and corrected by internal standard.

#### Probe recovery

Probe recovery was determined using an *in vitro* method previously described by Li et al. [51] Probes were immersed in a Petri dish filled with a predetermined concentration of DOX and DOXol and perfused with Ringer’s solution. Probe recovery was determined by dividing the concentration of DOX and DOXol in the recovered dialysate by the concentrations in the Petri dish. A flow rate of 3 μL/min was used in this experiment, yielding a 10 and 18% recovery rate of DOX and DOXol, respectively. It is important to note that, for the scope of this study, the stability of the environment surrounding the probe is critical as it allows for the constant collection of undisturbed concentrations of the drug and its metabolite. As such, collections were done in resting conditions and after a 60 minute period of equilibration.

#### Statistics

Changes in plasma, interstitial and muscle DOX and DOXol concentrations in comparison to baseline, which ultimately represents zero drug concentration, were analyzed using ANOVA and significance was accepted at P<0.05. Similarly, the differences between doses and each time points were analyzed using ANOVA where significance was accepted at P<0.05. When significant changes were observed in the comparisons, a Tukey’s *post hoc* test for multiple comparisons was used to determine where the significance occurred. All values used in graphical representations are displayed as mean ± SEM.

## References

[1] Minotti G, Menna P, Salvatorelli E, Cairo G, Gianni L. Anthracyclines: molecular advances and pharmacologic developments in antitumor activity and cardiotoxicity. Pharmacological reviews. 2004;56(2):185–229. Epub 2004/06/01. 10.1124/pr.56.2.6 . 15169927

[2] Momparler RL, Karon M, Siegel SE, Avila F. Effect of adriamycin on DNA, RNA, and protein synthesis in cell-free systems and intact cells. Cancer research. 1976;36(8):2891–5. Epub 1976/08/01. . 1277199

[3] Wang S, Konorev EA, Kotamraju S, Joseph J, Kalivendi S, Kalyanaraman B. Doxorubicin induces apoptosis in normal and tumor cells via distinctly different mechanisms. intermediacy of H(2)O(2)- and p53-dependent pathways. The Journal of biological chemistry. 2004;279(24):25535–43. 10.1074/jbc.M400944200 . 15054096

[4] Olson RD, Mushlin PS, Brenner DE, Fleischer S, Cusack BJ, Chang BK, et al Doxorubicin cardiotoxicity may be caused by its metabolite, doxorubicinol. Proceedings of the National Academy of Sciences of the United States of America. 1988;85(10):3585–9. Epub 1988/05/01. 2897122 10.1073/pnas.85.10.3585 PMC280258

[5] Minotti G, Recalcati S, Mordente A, Liberi G, Calafiore AM, Mancuso C, et al The secondary alcohol metabolite of doxorubicin irreversibly inactivates aconitase/iron regulatory protein-1 in cytosolic fractions from human myocardium. FASEB journal: official publication of the Federation of American Societies for Experimental Biology. 1998;12(7):541–52. Epub 1998/05/12. . 9576481 10.1096/fasebj.12.7.541

[6] Boucek RJ Jr., Olson RD, Brenner DE, Ogunbunmi EM, Inui M, Fleischer S. The major metabolite of doxorubicin is a potent inhibitor of membrane-associated ion pumps. A correlative study of cardiac muscle with isolated membrane fractions. The Journal of biological chemistry. 1987;262(33):15851–6. . 2890636

[7] Hayward R, Hydock D, Gibson N, Greufe S, Bredahl E, Parry T. Tissue retention of doxorubicin and its effects on cardiac, smooth, and skeletal muscle function. Journal of physiology and biochemistry. 2013;69(2):177–87. 10.1007/s13105-012-0200-0 . 22890792

[8] Gilliam LA, St Clair DK. Chemotherapy-induced weakness and fatigue in skeletal muscle: the role of oxidative stress. Antioxidants & redox signaling. 2011;15(9):2543–63. 10.1089/ars.2011.3965 21457105 PMC3176345

[9] van Norren K, van Helvoort A, Argiles JM, van Tuijl S, Arts K, Gorselink M, et al Direct effects of doxorubicin on skeletal muscle contribute to fatigue. British journal of cancer. 2009;100(2):311–4. 10.1038/sj.bjc.6604858 19165199 PMC2634729

[10] Smuder AJ, Kavazis AN, Min K, Powers SK. Exercise protects against doxorubicin-induced oxidative stress and proteolysis in skeletal muscle. J Appl Physiol (1985). 2011;110(4):935–42. 10.1152/japplphysiol.00677.2010 21310889 PMC3075128

[11] Yu AP, Pei XM, Sin TK, Yip SP, Yung BY, Chan LW, et al Acylated and unacylated ghrelin inhibit doxorubicin-induced apoptosis in skeletal muscle. Acta physiologica. 2014;211(1):201–13. 10.1111/apha.12263 . 24581239

[12] Smuder AJ, Kavazis AN, Min K, Powers SK. Exercise protects against doxorubicin-induced markers of autophagy signaling in skeletal muscle. J Appl Physiol (1985). 2011;111(4):1190–8. 10.1152/japplphysiol.00429.2011 . 21778418

[13] MacLean DA, Sinoway LI, Leuenberger U. Systemic hypoxia elevates skeletal muscle interstitial adenosine levels in humans. Circulation. 1998;98(19):1990–2. Epub 1998/11/10. . 9808594 10.1161/01.cir.98.19.1990

[14] MacLean DA, Bangsbo J, Saltin B. Muscle interstitial glucose and lactate levels during dynamic exercise in humans determined by microdialysis. J Appl Physiol (1985). 1999;87(4):1483–90. Epub 1999/10/12. . 10517782 10.1152/jappl.1999.87.4.1483

[15] Leuenberger UA, Johnson D, Loomis J, Gray KS, MacLean DA. Venous but not skeletal muscle interstitial nitric oxide is increased during hypobaric hypoxia. European journal of applied physiology. 2008;102(4):457–61. Epub 2007/11/07. 10.1007/s00421-007-0601-x . 17985154

[16] Delgado JM, DeFeudis FV, Roth RH, Ryugo DK, Mitruka BM. Dialytrode for long term intracerebral perfusion in awake monkeys. Archives internationales de pharmacodynamie et de therapie. 1972;198(1):9–21. Epub 1972/01/01. . 4626478

[17] Teraoka K, Hirano M, Yamaguchi K, Yamashina A. Progressive cardiac dysfunction in adriamycin-induced cardiomyopathy rats. European journal of heart failure. 2000;2(4):373–8. Epub 2000/12/13. . 11113713 10.1016/s1388-9842(00)00111-2

[18] Jensen RA, Acton EM, Peters JH. Doxorubicin cardiotoxicity in the rat: comparison of electrocardiogram, transmembrane potential, and structural effects. Journal of cardiovascular pharmacology. 1984;6(1):186–200. Epub 1984/01/01. . 6199603

[19] Yen HC, Oberley TD, Vichitbandha S, Ho YS, St Clair DK. The protective role of manganese superoxide dismutase against adriamycin-induced acute cardiac toxicity in transgenic mice. The Journal of clinical investigation. 1996;98(5):1253–60. Epub 1996/09/01. 10.1172/JCI118909 8787689 PMC507548

[20] Guerra J, De Jesus A, Santiago-Borrero P, Roman-Franco A, Rodriguez E, Crespo MJ. Plasma nitric oxide levels used as an indicator of doxorubicin-induced cardiotoxicity in rats. The hematology journal: the official journal of the European Haematology Association / EHA. 2005;5(7):584–8. Epub 2005/02/05. 10.1038/sj.thj.6200573 . 15692604

[21] Iqbal M, Dubey K, Anwer T, Ashish A, Pillai KK. Protective effects of telmisartan against acute doxorubicin-induced cardiotoxicity in rats. Pharmacological reports: PR. 2008;60(3):382–90. Epub 2008/07/16. . 18622063

[22] Nagi MN, Mansour MA. Protective effect of thymoquinone against doxorubicin-induced cardiotoxicity in rats: a possible mechanism of protection. Pharmacological research: the official journal of the Italian Pharmacological Society. 2000;41(3):283–9. Epub 2000/02/17. 10.1006/phrs.1999.0585 . 10675279

[23] Arola OJ, Saraste A, Pulkki K, Kallajoki M, Parvinen M, Voipio-Pulkki LM. Acute doxorubicin cardiotoxicity involves cardiomyocyte apoptosis. Cancer research. 2000;60(7):1789–92. Epub 2000/04/15. . 10766158

[24] Thorn CF, Oshiro C, Marsh S, Hernandez-Boussard T, McLeod H, Klein TE, et al Doxorubicin pathways: pharmacodynamics and adverse effects. Pharmacogenetics and genomics. 2011;21(7):440–6. Epub 2010/11/05. 10.1097/FPC.0b013e32833ffb56 21048526 PMC3116111

[25] Mordente A, Meucci E, Silvestrini A, Martorana GE, Giardina B. New developments in anthracycline-induced cardiotoxicity. Current medicinal chemistry. 2009;16(13):1656–72. Epub 2009/05/16. . 19442138 10.2174/092986709788186228

[26] Kassner N, Huse K, Martin HJ, Godtel-Armbrust U, Metzger A, Meineke I, et al Carbonyl reductase 1 is a predominant doxorubicin reductase in the human liver. Drug metabolism and disposition: the biological fate of chemicals. 2008;36(10):2113–20. Epub 2008/07/19. 10.1124/dmd.108.022251 . 18635746

[27] Bains OS, Karkling MJ, Lubieniecka JM, Grigliatti TA, Reid RE, Riggs KW. Naturally occurring variants of human CBR3 alter anthracycline in vitro metabolism. The Journal of pharmacology and experimental therapeutics. 2010;332(3):755–63. Epub 2009/12/17. 10.1124/jpet.109.160614 . 20007405

[28] Heibein AD, Guo B, Sprowl JA, Maclean DA, Parissenti AM. Role of aldo-keto reductases and other doxorubicin pharmacokinetic genes in doxorubicin resistance, DNA binding, and subcellular localization. BMC cancer. 2012;12:381 Epub 2012/09/04. 10.1186/1471-2407-12-381 22938713 PMC3495881

[29] Mahnik SN, Rizovski B, Fuerhacker M, Mader RM. Development of an analytical method for the determination of anthracyclines in hospital effluents. Chemosphere. 2006;65(8):1419–25. 10.1016/j.chemosphere.2006.03.069. 16713616

[30] van Asperen J, van Tellingen O, Tijssen F, Schinkel AH, Beijnen JH. Increased accumulation of doxorubicin and doxorubicinol in cardiac tissue of mice lacking mdr1a P-glycoprotein. British journal of cancer. 1999;79(1):108–13. Epub 1999/07/17. 10.1038/sj.bjc.6690019 10408701 PMC2362153

[31] Shin M, Matsunaga H, Fujiwara K. Differences in accumulation of anthracyclines daunorubicin, doxorubicin and epirubicin in rat tissues revealed by immunocytochemistry. Histochemistry and cell biology. 2010;133(6):677–82. Epub 2010/04/29. 10.1007/s00418-010-0700-3 . 20424853

[32] Sacco G, Giampietro R, Salvatorelli E, Menna P, Bertani N, Graiani G, et al Chronic cardiotoxicity of anticancer anthracyclines in the rat: role of secondary metabolites and reduced toxicity by a novel anthracycline with impaired metabolite formation and reactivity. British journal of pharmacology. 2003;139(3):641–51. Epub 2003/06/06. 10.1038/sj.bjp.0705270 12788824 PMC1573869

[33] Ciaccio M, Valenza M, Tesoriere L, Bongiorno A, Albiero R, Livrea MA. Vitamin A inhibits doxorubicin-induced membrane lipid peroxidation in rat tissues in vivo. Archives of biochemistry and biophysics. 1993;302(1):103–8. Epub 1993/04/01. 10.1006/abbi.1993.1186 . 8470886

[34] Arnold RD, Slack JE, Straubinger RM. Quantification of Doxorubicin and metabolites in rat plasma and small volume tissue samples by liquid chromatography/electrospray tandem mass spectroscopy. Journal of chromatography B, Analytical technologies in the biomedical and life sciences. 2004;808(2):141–52. Epub 2004/07/21. 10.1016/j.jchromb.2004.04.030 15261807 PMC2896316

[35] Doroshow JH, Tallent C, Schechter JE. Ultrastructural features of Adriamycin-induced skeletal and cardiac muscle toxicity. The American journal of pathology. 1985;118(2):288–97. Epub 1985/02/01. 3970141 PMC1887878

[36] Gibson NM, Quinn CJ, Pfannenstiel KB, Hydock DS, Hayward R. Effects of age on multidrug resistance protein expression and doxorubicin accumulation in cardiac and skeletal muscle. Xenobiotica; the fate of foreign compounds in biological systems. 2013 Epub 2013/10/22. 10.3109/00498254.2013.846489 . 24138210

[37] Skovsgaard T, Nissen NI. Membrane transport of anthracyclines. Pharmacology & therapeutics. 1982;18(3):293–311. Epub 1982/01/01. 10.1016/j.exer.2015.09.002 6762567

[38] Awasthi S, Singhal SS, Awasthi YC, Martin B, Woo JH, Cunningham CC, et al RLIP76 and Cancer. Clinical cancer research: an official journal of the American Association for Cancer Research. 2008;14(14):4372–7. Epub 2008/07/17. 10.1158/1078-0432.CCR-08-0145 18628450 PMC2919285

[39] Georges E, Bradley G, Gariepy J, Ling V. Detection of P-glycoprotein isoforms by gene-specific monoclonal antibodies. Proceedings of the National Academy of Sciences of the United States of America. 1990;87(1):152–6. Epub 1990/01/01. 1688652 10.1073/pnas.87.1.152 PMC53218

[40] Mushlin PS, Cusack BJ, Boucek RJ Jr., Andrejuk T, Li X, Olson RD. Time-related increases in cardiac concentrations of doxorubicinol could interact with doxorubicin to depress myocardial contractile function. British journal of pharmacology. 1993;110(3):975–82. Epub 1993/11/01. 8298821 10.1111/j.1476-5381.1993.tb13909.x PMC2175809

[41] Cusack BJ, Young SP, Driskell J, Olson RD. Doxorubicin and doxorubicinol pharmacokinetics and tissue concentrations following bolus injection and continuous infusion of doxorubicin in the rabbit. Cancer chemotherapy and pharmacology. 1993;32(1):53–8. Epub 1993/01/01. . 8462124 10.1007/BF00685876

[42] Peters JH, Gordon GR, Kashiwase D, Acton EM. Tissue distribution of doxorubicin and doxorubicinol in rats receiving multiple doses of doxorubicin. Cancer chemotherapy and pharmacology. 1981;7(1):65–9. Epub 1981/01/01. . 7340990 10.1007/BF00258216

[43] Lim S, Shin JY, Jo A, Jyothi KR, Nguyen MN, Choi TG, et al Carbonyl reductase 1 is an essential regulator of skeletal muscle differentiation and regeneration. The international journal of biochemistry & cell biology. 2013;45(8):1784–93. Epub 2013/06/05. 10.1016/j.biocel.2013.05.025 . 23732109

[44] Bloemberg D, Quadrilatero J. Rapid determination of myosin heavy chain expression in rat, mouse, and human skeletal muscle using multicolor immunofluorescence analysis. PloS one. 2012;7(4):e35273 Epub 2012/04/25. 10.1371/journal.pone.0035273 22530000 PMC3329435

[45] Goormaghtigh E, Huart P, Praet M, Brasseur R, Ruysschaert JM. Structure of the adriamycin-cardiolipin complex. Role in mitochondrial toxicity. Biophysical chemistry. 1990;35(2–3):247–57. Epub 1990/04/01. . 2204444 10.1016/0301-4622(90)80012-v

[46] Anderson AB, Xiong G, Arriaga EA. Doxorubicin accumulation in individually electrophoresed organelles. Journal of the American Chemical Society. 2004;126(30):9168–9. Epub 2004/07/30. 10.1021/ja0492539 15281791

[47] Erzen I, Janacek J, Kubinova L. Characterization of the capillary network in skeletal muscles from 3D data. Physiological research / Academia Scientiarum Bohemoslovaca. 2011;60(1):1–13. Epub 2010/10/16. . 20945967 10.33549/physiolres.931988

[48] Murakami S, Fujino H, Takeda I, Momota R, Kumagishi K, Ohtsuka A. Comparison of capillary architecture between slow and fast muscles in rats using a confocal laser scanning microscope. Acta medica Okayama. 2010;64(1):11–8. Epub 2010/03/05. . 20200579 10.18926/AMO/32859

[49] Hellsten Y, Maclean D, Radegran G, Saltin B, Bangsbo J. Adenosine concentrations in the interstitium of resting and contracting human skeletal muscle. Circulation. 1998;98(1):6–8. Epub 1998/07/17. . 9665052 10.1161/01.cir.98.1.6

[50] MacLean DA, Vickery LM, Sinoway LI. Elevated interstitial adenosine concentrations do not activate the muscle reflex. American journal of physiology Heart and circulatory physiology. 2001;280(2):H546–53. Epub 2001/02/13. . 11158950 10.1152/ajpheart.2001.280.2.H546

[51] Li J, King NC, Sinoway LI. ATP concentrations and muscle tension increase linearly with muscle contraction. J Appl Physiol (1985). 2003;95(2):577–83. Epub 2003/04/30. 10.1152/japplphysiol.00185.2003 . 12716867

